# Characterization, stability and antioxidant activity of curcumin nanocomplexes with soy protein isolate and pectin

**DOI:** 10.1016/j.crfs.2023.100530

**Published:** 2023-06-07

**Authors:** Lijuan Fu, Suo Tan, Ruiru Si, Yueyue Qiang, Hang Wei, Biao Huang, Mengzhu Shi, Ling Fang, Jianwei Fu, Shaoxiao Zeng

**Affiliations:** a Institute of Quality Standards & Testing Technology for Agro-products, Fujian Academy of Agricultural Sciences/Fujian Key Laboratory of Agro-products Quality and Safety, Fuzhou, 350003, China; b College of Food Science, Fujian Agriculture and Forestry University, Fuzhou, 350002, China

**Keywords:** Curcumin, Soy protein isolate, Pectin, Nanocomplexes, Antioxidant activity

## Abstract

Curcumin (Cur) has antioxidant, anti-inflammatory and other biological activities, but its poor stability, low water solubility and other defects limit the application. Herein, Cur was nanocomposited with soy isolate protein (SPI) and pectin (PE) for the first time and its characterization, bioavailability and antioxidant activity were discussed. The optimal encapsulation process of SPI-Cur-PE was as follow: the addition amount of PE was 4 mg, Cur was 0.6 mg and at pH of 7. It was observed by SEM that SPI-Cur-PE were partially aggregated. The average particle size of SPI-Cur-PE was 210.1 nm and the zeta potential was −31.99 mV. Through XRD, FT-IR and DSC analysis, the SPI-Cur-PE was formed through hydrophobic interaction and electrostatic interaction. The SPI-Cur-PE released more slowly in simulated gastrointestinal treatment and displayed higher photostability and thermal stability. SPI-Cur-PE, SPI-Cur and free Cur had scavenging activities for 2,2′-azino-bis (3-ethylbenzothiazoline-6-sulfonic acid) (ABTS) and 1,1-diphenyl-2-picryl-hydrazyl (DPPH) radicals.

## Highlights

• The SPI-Cur-PE nanocomplexes were prepared successfully for the first time.
• The thermal stability and photostability of SPI-Cur-PE were higher than that of free Cur.
• SPI-Cur-PE shown lower releasing speed than SPI-Cur and free Cur in vitro release test.
• The release rate of SPI-Cur-PE in SGF was lower than that of SPI-Cur and free Cur.
• The SPI-Cur-PE represented significantly scavenging activity of ABTS and DPPH radicals.

## Graphical abstract

**Figure:**
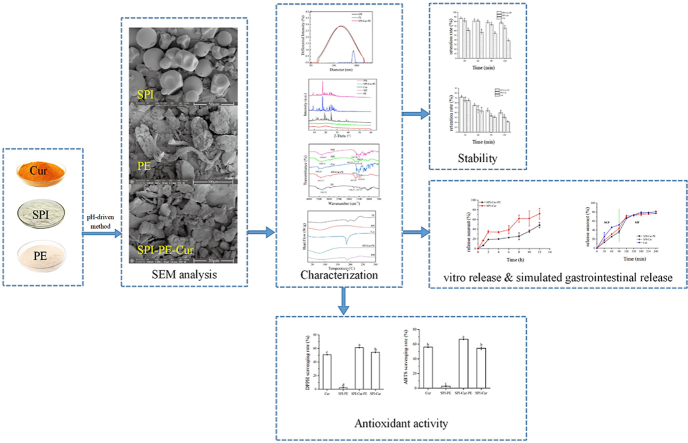

## 1 Introduction

Cur is a polyphenolic compound extracted from dried rhizome of Curcuma *longa* L. It is a kind of resource, which has been traditionally used both as medicine and food. It has broad range of activities in preventing or curing various diseases such as relieving pain, removing stasis, relieving depression, cholagogue, sedative, promote digestion and remove stagnated food for centuries in China (Liu et al., 2020). Meanwhile, it is widely used for a variety of food coloring. The modern research has found that Cur has anti-cancer, anti-inflammatory, antibacterial, antioxidant, lipid-lowering and other biological activities (Sanidad et al., 2019). Research had found that Cur could enhance the activity of antioxidant enzymes, such as catalase, superoxide dismutase, glutathione peroxidase (Soleimani et al., 2018). Abdel-Daim et al. found that 200 mg/kg Cur could improve the total antioxidant activity of the liver of rats with oxidative stress (Abdel-Daim and Abdou, 2015). Cur could show potent anticancer effects through regulating mechanisms such as cell cycle arrest of apoptosis, cell migration and invasion of cancer cell lines (Cheng et al., 2001; Momtazi et al., 2016). Furthermore, Cur could protect against acute lung injury, liver-kidney oxidative damage and cardiac function damage in rats by inhibiting inflammatory cytokines (J. Wang et al., 2015). In summary, the functional activities based on Cur have potential application prospects in food, medicine industries.

However, Cur is sensitive to temperature, oxygen and light, and it has poor stability. Its functional activities are limited due to the sensitive, lower water solubility and low bioavailability (Ma et al., 2019). Studies had shown that constructing a carrier system to encapsulate the Cur was an effective way to lessen these limitations (Jiang et al., 2020). Carrier system refers to the use of macromolecular substances (such as proteins, polysaccharides, phospholipids, etc.) to interact with active substances. The active substances could be encapsulated or combined into these macromolecular substances through hydrophobic, hydrogen bonding, electrostatic force and other ways, so as to improve the stability and biological utilization of active components (W. Huang et al., 2020). At present, nanocomplexes, liposomes, nanoemulsions and other carrying systems are commonly used to improve the water solubility of Cur in food field, which prevent its degradation in the microenvironment and enhance the slow-release efficacy (Mirpoor et al., 2017).

SPI has been widely used in the food industry because of its great emulsification, foaming, gelling, film forming and other functional characteristics. The research had shown that encapsulating Cur in the protein carrier system could effectively improve the water solubility, stability and bioavailability (Y. Zhang et al., 2019). However, the protein carrier system was prone to flocculation and precipitation when the environmental pH was close to the isoelectric point or the salt concentration was high (S. Wang et al., 2020). Binding with anionic polysaccharide could improve the spatial stability of protein (Xu et al., 2020). It was a better way to improve the water solubility and stability of Cur by using polysaccharides and protein to encapsulate Cur, which could improve the transport characteristics of proteins and the stability of bioactive components (Mahmood et al., 2016). Chen et al. found that the solubility of Cur-SPI in water has increased more than 98 times compared to free Cur in water, and the formation of nanocomposites had greatly improved the storage stability (F. Chen et al., 2015). Mirpoor et al. found that the *β*-lactoglobulin (BLG)-sodium alginate (ALG) electrostatic nanocomplex encapsulated and transferred Cur in a transparent acidic medium, with the result that its physical stability was significantly improved (Mirpoor et al., 2017). Moreover, the nanocomplexes had a considerable protective effect on thermal degradation of Cur, improving the bioavailability of Cur. Li et al. prepared core-shell biopolymer nanoparticles for encapsulation and delivery of Cur, which enhanced DPPH radical scavenging activity and prolonged gastrointestinal release time (Li et al., 2021).

Based on the above, the primary goal of this research was intended to elucidate the nanocomplexes of Cur with SPI and PE on the stability and antioxidant activity. The pH-driven method was used to construct the Cur transport carrier with SPI as the main molecule, and PE was added for wall material to form and characterize the SPI-Cur-PE nanocomplexes. The physical stability of SPI-Cur-PE was studied mainly through thermal and photostability experiments, and free radical scavenging experiments were conducted on SPI-Cur-PE. It also determined the release of SPI-Cur-PE in vitro and simulated gastrointestinal, and confirmed that Cur bioavailability could be improved.

## 2 Materials and methods

### 2.1 Chemicals and materials

Cur (Reference Standards, 99%) were purchased from Tan Ink Quality Inspection-Standard Material Center (Changzhou, China). SPI (Food-Grade) was purchased from Duly Reagent Co., Ltd (Nanjing, China). PE (Food Grade), Trypsin, Pepsin (>3,000 U/mg) and pig bile salt were purchased from Macklin Biotech Co., Ltd (Shanghai, China). NaOH, NaCl and absolute ethyl alcohol (Analytical Reagent) were purchased from Sinopharm Chemical Reagent Co., Ltd (Shanghai, China). DPPH and ABTS (Reference Standards) were purchased from Shanghai Yingxin Laboratory Equipment Co., Ltd (Shanghai, China).

### 2.2 Preparation of the SPI-Cur-PE nanocomplexes

According to the method of Dias et al. (Dias et al., 2020), SPI solution with mass concentration of 1 mg/mL was prepared by deionized water. After magnetic stirring for 2 h, 1 M NaOH was added to adjust pH 7.0. The mixture was centrifugated at 5,000 r/min for 5 min to remove insoluble matters, and the obtained solution was pre-treatment SPI solution. The PE solution with a certain concentration was prepared by deionized water and stirred with magnetic force at 70 °C for 40 min. The Cur solution with certain concentration (Different amounts of Cur added (0.4, 0.6 and 0.8 mg) in the single factor experiment corresponded to different Cur concentrations, then the corresponding concentration of Cur was 0.04 mg/mL) was prepared by absolute ethanol. SPI-Cur-PE nanocomplexes were prepared by pH-driven method. The Cur solution was added dropwise to the pretreated SPI solution according to the volume ratio of 1:10 (Cur: SPI) (pre-experiment was conducted to determine the volume ratio as shown in supplement Fig. 1a), stirred magnetically at 500 r/min and adjusted the pH to 10 with NaOH. Then the pH of the solutions was adjusted to 7.0 with HCl and stirred magnetically at 7500 r/min for 2 h. Afterwards the PE solution was dropwise added to the solution with the volume ratio of 1:1 (SPI: PE) and magnetic stirring at 500 r/min for 2 h. The solution was extracted by centrifugal force at 5,000 r/min for 5 min and removed the precipitation. The supernatant was freeze-dried to prepare SPI-Cur-PE nanocomplexes.

The physical mixture of Cur, SPI, and PE (physical mixture, PM) was prepared as a control group. It was manually ground with a mortar and pestle for 15 min to allow for a uniform mixing of Cur, SPI, and PE.

### 2.3 Determination of the encapsulation rate

The prepared SPI-Cur-PE nanocomplexes was mixed with absolute ethanol, vortexed with centrifugation at 5,000 r/min for 5 min to extract Cur. The supernatant was taken, and the sediment was added to absolute ethanol. The extraction was repeated until the supernatant became colorless to determine the Cur content in the nanocomplexes. Determination of Cur was performed by high performance liquid chromatograph (HPLC, SPD-M40, SHIMADZU) with C18 column (250 × 4.6 mm, 5 μm, Welch Materials).

The detector conditions were followed the description used by Qiang et al. (2021). The standard curve of Cur was plotted with the peak area as the vertical axis and the corresponding concentration as the horizontal axis. The concentration of Cur in nanocomplexes was determined according to the standard curve. The Cur encapsulation rate was calculated using the following formula.

(1) $$Encapsulation\;rate\left(\%\right)=\frac{Measured\;quantity\;of\;Cur\;in\;nanocomplexes}{Cur\;addition}\times 100\%$$

### 2.4 The single factor experiment and orthogonal experiment

The effects of PE addition (2, 4, 6, 8 and 10 mg), Cur addition (0.4, 0.6, 0.8, 1.0 and 1.2 mg), and pH (5, 6, 7, 8, 9 and 10) on the SPI-Cur-PE nanocomplexes encapsulation rate were investigated, respectively, and the optimal level was screened for orthogonal experiment.

According to the single factor experiment results, the L_9_ (3^4^) orthogonal experiment table was designed to investigate the effect of PE addition, Cur addition and the pH on the encapsulation rate of SPI-Cur-PE nanocomplexes, so as to obtain the optimal encapsulation process of nanocomplexes.

### 2.5 The SPI-Cur-PE nanocomplexes characterization

#### 2.5.1 Scanning electron microscopy (SEM)

The SEM produced images of the SPI-Cur-PE nanocomplexes by scanning the surface with a focused electron beam and it was performed according to the method described By Zhang et al. (Y. Zhang et al., 2019). The surface morphology of the SPI, PE, and SPI-Cur-PE nanocomplexes was observed at an accelerating voltage of 15.0 kV at room temperature using the scanning electron microscope (JSM-6380LV, JEOL Ltd., Japan). Prior to testing, the cropped samples were fixed on a copper stage with conductive glue, then sprayed with gold foil, gold-plated the samples in a vacuum gold plating machine, and the microstructure of the different samples was observed under a scanning electron microscope.

#### 2.5.2 Zeta-potential and particle size

The particle size and Zeta-potential of SPI, PE and SPI-Cur-PE were analyzed by using Zeta-potential & Particle size Analyzer (NanoPlus3, Micromeritics, U.S.A) according to the method proposed by Zhan et al. (Zhan et al., 2020). The sample particle size was measured on a 633 nm, 25 °C, 173° backscattering detector, and the sample interface surface charge was measured by the dynamic light scattering to obtain the Zeta potential.

#### 2.5.3 X-Ray diffraction (XRD)

The procedure of XRD analysis was previously described by Celebioglu, & Uyar (2020). The copper target X-ray tubes were used to obtain XRD profiles of free Cur, SPI, PE, SPI-Cur-PE nanocomplexes, and PM at accelerating voltage of 40 kV, current intensity of 40 mA, wavelength of 0.1546 nm, scan speed of 2°/min, scan range of 2θ = 3°–60°.

#### 2.5.4 Fourier transform infrared spectroscopy (FT-IR)

FT-IR analysis was obtained by the method proposed by Liu et al. (Liu et al., 2019). The Cur, SPI, PE, SPI-Cur-PE nanocomplexes and PM were mixed with potassium bromide at 1:100 (w/w), respectively, and then pressed as samples to be tested. Tests were performed under the determination parameters with scan range of 4,000 cm^−1^ - 400 cm^−1^ and a resolution of 4 cm^−1^.

#### 2.5.5 Differential scanning calorimetry (DSC)

DSC is a widely used thermal analysis method, after the formation of a new inclusion compound, the boiling point, the melting point of the original material, and the biochemical point will change. According to the method proposed by Wang et al. (Wang et al., 2019, Wang et al., 2019), DSC signals for free Cur, SPI, PE, SPI-Cur-PE nanocomplexes and PM were obtained by simultaneous thermal analyzer (STA449F5, NETZSCH-Gerätebau GmbH, Germany). Placed each sample (2–5 mg) in a closed aluminum plate in a sealed aluminum pan, with the empty sealed pan as the reference. Assays were performed at scan speed of 10 °C/min and with a scan range of 30°C–300 °C.

### 2.6 Stability of SPI-Cur-PE nanocomplexes

#### 2.6.1 Thermal stability

The thermal stability experiment were carried out based on the methods used in a previous study (W. Huang et al., 2020). Free Cur, SPI-Cur, SPI-Cur-PE nanocomplexes (The concentration of Cur was equal) were placed in an 85 °C constant water bath for 30, 60, 90 and 120 min before immediately cooling to room temperature. The absorbance of free Cur, SPI-Cur, SPI-Cur-PE nanocomplexes were measured at 426 nm. The light absorption value of freshly prepared Cur solutions was recorded as A_0_, and at other time points as A_i_. The retention rate of Cur was calculated according to the following formula.

(2) $$Cur\;retention\;rate\left(\%\right)=\frac{A_{i}}{A_{0}}\times 100\%$$

#### 2.6.2 Photostability

Solutions of free Cur, SPI-Cur, and SPI-Cur-PE nanocomplexes were placed in 25 °C, 100 W/m^2^ light addition and underwent 30, 60, 90 and 120 min light treatment according to the method reported by Zhan et al. (Zhan et al., 2020). The absorbance value at 426 nm in the solution was indicated. The absorption value of fresh prepared Cur solution was recorded as A_0_, the absorption value of other time points was recorded as A_i_, and the retention rate of Cur was calculated according to formula (2).

### 2.7 In vitro release

The in vitro release was performed by a modification of the previously described procedure (Guzman-Villanueva et al., 2013). SPI-Cur, SPI-Cur-PE were dissolved in phosphate buffered saline (PBS, 0.01 M, pH 7.4) and placed in 37 °C oscillation water bath pot of 150 r/min, taken out 1 mL for centrifugation after 1, 2, 4, 6, 8, 10, and 12 h, respectively. 0.5 mL of supernatant was redissolved in 1 mL methanol, and 20 μL of solution was injected into HPLC (SPD-M40, Shimadzu) to determine the Cur release at different time intervals.

### 2.8 Simulated gastrointestinal digestion

To observe the effect of SPI-Cur-PE nanocomplexes releasing Cur in simulated gastrointestinal digestion, existing method (H. Zhang et al., 2021) was adopted and modified for the experiment. Preparation of simulated gastric fluid (SGF): 3.2 mg/mL of pepsin solution was configured and adjusted the solution pH to 2.0. Preparation of simulation of intestinal fluid (SIF): the solution was configured with 6.8 mg/mL K_2_HPO_4_, 0.8 mg/mL pancreatic enzyme, 20 mg/mL pig bile salt extract, 8.775 mg/mL NaCl and regulated pH to 7.4.

Simulated gastrointestinal processes: 50 mL of newly prepared free Cur, SPI-Cur, and SPI-Cur-PE nanocomplexes solutions were mixed with 50 mL SGF solution and placed in a 37 °C shaker with 100 rpm/min for 90 min. 2 mL samples were collected at 30 min, 60 min, and 90 min. 2 mL SGF solution was added after sampling. The digestion solution was added to 100 mL SIF solution after 90 min, and then the mixture was placed at 37 °C, 100 rpm/min for 150 min. 2 mL samples were collected every 30 min intervals. 2 mL SIF solution was added after sampling. All collected samples were centrifuged for 5 min at 5,000 r/min to remove insoluble fractions, and then released to determine the absorbance of solution at 426 nm.

### 2.9 Antioxidant activity in vitro

#### 2.9.1 The DPPH scavenging activity

The DPPH assay was described in the previous paper (Ak and Gülçin, 2008). 1 mL free Cur, SPI-PE, SPI-Cur, SPI-Cur-PE solution were mixed with 1 mL DPPH solution in the dark at room temperature for 30 min. The absorbance was measured at 517 nm and the absorption value was recorded as A_i_. 1 mL distilled water was mixed with 1 mL DPPH solution as a blank control, the absorption value was recorded as A_0_, and the DPPH scavenging rate was calculated according to formula (3).

(3) $$DPPH\;scavenging\;rate\;\left(\%\right)=\frac{A_{0}-A_{i}}{A_{0}}\times 100\%$$

#### 2.9.2 The ABTS scavenging activity

The ABTS assay was performed according to the method proposed by Ak and Gülçin (2008). ABTS was dissolved in deionized water to the concentration of 7 mM. Potassium persulfate at the concentration of 2.45 mM was dissolved in ABTS solution and stored in dark for 12 h as stock solution. The ABTS stock solution was diluted with 5 mM potassium phosphate buffer solution (pH 7.4) so that its absorbance at 734 nm was 0.75–0.80, which was used as the ABTS working liquor.

1 mL free Cur, SPI-PE, SPI-Cur, SPI-Cur-PE solutions were mixed with ABTS working liquor, reacted and avoided light for 10 min at room temperature. The absorbance was measured at 734 nm and the absorbent value was A_i_. 1 mL distilled water was fully mixed with 1 mL ABTS working solution and shaken as blank control, the absorbent value was recorded as A_0_. The ABTS scavenging rate was calculated by formula (4).

(4) $$ABTS\;scavenging\;rate\left(\%\right)=\frac{A_{0}-A_{i}}{A_{0}}\times 100\%$$

### 2.10 Statistical analysis

All experiments were independently in triplicate and results were averaged. Drawing was performed with GraphPad Prism 7.0 and Origin 2019 software. One-way ANOVA under Duncan's multiple range test and independent-samples *t*-test were used for statistical comparison. The values of *p* < 0.05 were considered as statistically significant differences.

## 3 Results and discussion

### 3.1 Results of one-factor and orthogonal test

Electrostatic interactions and hydrophobic interactions occurred between SPI and PE, and the addition ratio of the two could affect the composite efficiency of the nanocomplexes (Yuan et al., 2021). In addition, the water solubility of Cur is affected by pH, with high solubility in alkaline solutions, but unstable and is rapidly degraded into vanillin, ferulic acid and other substances after dissolution (Li et al., 2016; Liu et al., 2016; Rafiee et al., 2019), and the pH of the solution could also affect the SPI and PE solubility (S. Wang et al., 2020). Moreover, excessive Cur had adverse effects on the dispersion system (F. Chen et al., 2020, Chen et al., 2020). Through the single factor experiment and orthogonal experiment, Cur encapsulation rate as evaluation index, the optimal process conditions (seen supplement table 1) were finally determined as follow: the amount of PE addition was 4 mg, Cur addition was 0.6 mg, and pH value was 7.0, and the encapsulation rate of SPI-Cur-PE nanocomplexes prepared under this process condition was 97.81%.

### 3.2 Characterization of nanocomplexes

#### 3.2.1 SEM analysis

**Fig. 1:**
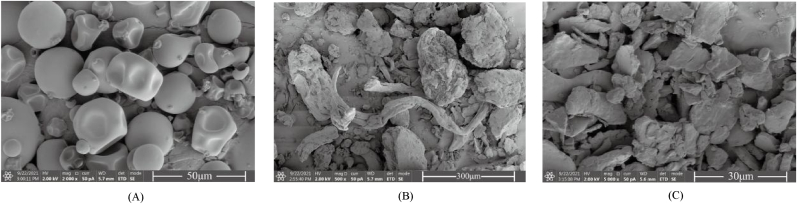
SEM analysis of SPI (A), PE (B) and SPI-Cur-PE nanocomplexes (C).

SEM is a qualitative technique used to observe the surface structure of raw materials or prepared preparations. The morphological features of the SPI, PE, and SPI-Cur-PE nanocomplexes were observed by SEM, and the results were shown in Fig. 1. The morphology of the SPI was uneven spherical, smooth surface and cluster, which was similar to previous studies (G. Huang et al., 2012). The morphology of PE presented block structure, the particle size of PE was uneven and had low density. Whereas, compared with the original morphology of SPI and PE, the morphology of SPI-Cur-PE nanocomplexes changed significantly. It presented irregular particle size block structure, and the agglomeration phenomenon was more obvious. This particle morphological change may be due to the interaction of Cur, SPI with PE (R. Wang et al., 2019). Or possibly because the SPI-Cur-PE nanocomplexes was freeze-dried during the preparation process, resulting in water loss, and thus the agglomeration phenomenon occurred (Sari et al., 2015).

#### 3.2.2 Zeta-potential and particle size analysis

**Fig. 2:**
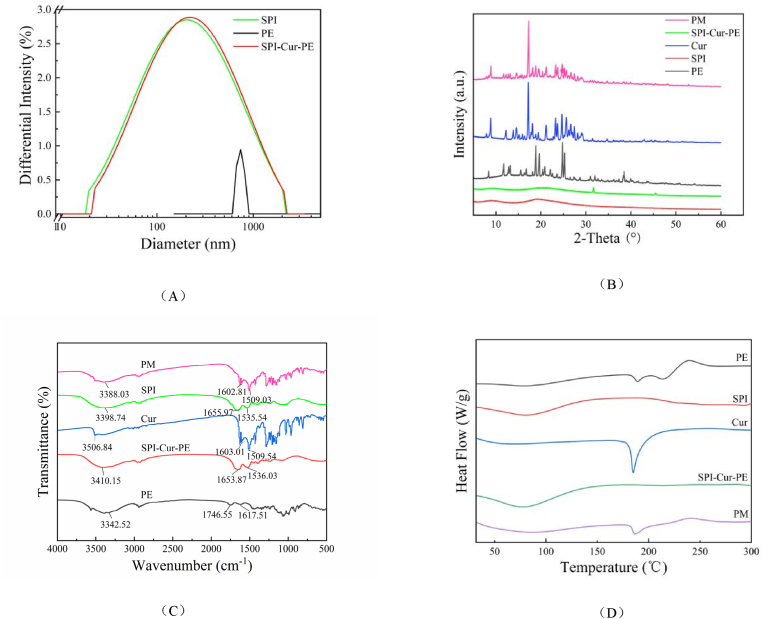
Characterization of the nanocomplexes.

The surface charge and particle size of the nanocomplexes have important effects on their particle stability. The particle size distribution of the SPI, PE, and SPI-Cur-PE nanocomplexes were examined, as shown in Fig. 2 (A), and the mean particle size and zeta potential were shown in Table 1. As could be seen, the particle size distribution of SPI-Cur-PE was slightly larger than SPI, but much smaller than the average particle size of PE, it probably due to the SPI-Cur-PE preparation affecting the internal structure of SPI and PE, forming the dense nanocomplexes (Patel et al., 2010). The zeta-potential of SPI-Cur-PE nanocomplexes was −31.99 mV, while the zeta-potential of SPI and PE was −41.42 mV and −19.01 mV, respectively, which indicated that the binding force between them may be the electrostatic interaction (S. Chen et al., 2020). Generally, the dispersion system is stable when the absolute value of the zeta potential is greater than 30 mV. When it is less than 30 mV but greater than 20 mV, the dispersion system shows short-term stability. Therefore it could be concluded that the SPI-Cur-PE nanocomplexes system (−31.99 mV) were stable.

**Table 1:** Particle size distribution and Zeta-potential analysis of SPI, PE and SPI-Cur-PE nanocomplexes.

	SPI	PE	SPI-Cur-PE
Z-average diameter (nm)	197.2	79610.1	210.1
Zeta-potential (mV)	−41.42	−19.01	−31.99

#### 3.2.3 XRD analysis

The XRD profiles of the Cur, SPI, PE, PM, and SPI-Cur-PE nanocomplexes were drawn using XRD analysis to determine the physical state changes of Cur in the SPI-Cur-PE nanocomplexes, as shown in Fig. 2 (B). The SPI had gentle peak at 2-Theta of 19.7°, indicating an amorphous state. The XRD spectrum of Cur had obvious characteristic peaks at 8.9°, 12.3°, 14.5°, 17.2°, 21.2°, 23.3°, 24.7°, 25.7° and 29.0°, declaring that Cur was crystal structure. However, the above XRD characteristic peaks of SPI-Cur-PE had disappeared, which proclaimed the Cur crystal structure had changed to amorphous state (Ota et al., 2018) and illustrated the interacted of Cur, SPI and PE.

#### 3.2.4 FI-TR analysis

The molecular structures of SPI, PE, Cur, SPI-Cur-PE, and PM were investigated by FT-IR analysis to further investigate the interaction mode between Cur, SPI, and PE in the SPI-Cur-PE nanocomplexes. The results were shown in Fig. 2 (C). SPI had obvious absorption bands at 3398.74 cm^−1^ (O–H stretching vibration), 1655.97 cm^−1^ (amide I bond, C=O stretching vibration), and 1533.54 cm^−1^ (amide Ⅱ bond, –NH bending vibration) (S. Chen et al., 2020; G. Huang et al., 2012). The absorption peak of PE at 3342.52 cm^−1^ was generated by the O–H stretching vibration of the intramolecular or intermolecular hydrogen bonds. The absorption peak of 1746.55 cm^−1^ was generated by the stretching vibration of C=O (-COOR) of the ester bond formed by the alduronic acid. The peak at the 1617.51 cm^−1^ was generated by the asymmetric stretching vibration of the free carboxyl or carboxylate C=O (-COO) (Chang et al., 2017; Hu et al., 2015). The Cur had no characteristic peaks in the carbonyl group region (1800 cm^−1^-1650 cm^−1^), which declared that Cur existed as keto-enol tautomerism. The absorption peak at 3506.84 cm^−1^ corresponded to the stretching vibration of Ph-OH, and the absorption peak of 1628.09 cm^−1^ corresponded to C=O stretching vibration. Then the 1429.19 cm^−1^ was the bending vibration of alkene C–H. The 1283.24 cm^−1^ and 1028.01 cm^−1^ were respectively corresponding to the stretching vibration of arene C–O and C–O–C (Feng et al., 2020; Ghobadi et al., 2021; S. Wang et al., 2020). The infrared spectrum of SPI-Cur-PE was not significantly different from SPI proclaiming that the combination of the three does not involve amide bonds in the peptide chain. It was indirectly confirming the inference that the Cur was bound to interact with the hydrophobic region of SPI mainly through hydrophobic interactions. In contrast to the SPI, the amide I bond position of the SPI-Cur-PE was blue-shifted, due to an electrostatic interaction between the SPI and the PE (Luo et al., 2011). From the foregoing, these results indicated that the recombination of Cur with SPI and PE may arise through hydrophobic interactions and electrostatic interactions.

#### 3.2.5 DSC analysis

The DSC analysis of SPI, PE, Cur, SPI-Cur-PE nanocomplexes and PM was performed to study the properties of Cur in SPI-Cur-PE nanocomplexes varied with temperature, and the results were shown in Fig. 2 (D). The significant endothermic peak of Cur was observed at 180 °C, due to the melting of the Cur crystals, it indicated that Cur existed in the form of crystal structure (L. Wu et al., 2019). The endothermic peaks of the SPI and PE corresponded to temperature value of 78.0 °C, mainly due to water evaporation. Whereas the endothermic peak of the SPI-Cur-PE corresponded to a temperature of 80.2 °C, higher than the SPI and PE. FI-TR analysis results demonstrated that electrostatic interactions and other non-covalent interactions of the SPI-Cur-PE may exist during their formation. These interactions made it had higher phase transition temperature, thus improved the stability of SPI-Cur-PE nanocomplexes (H. Zhang et al., 2021). However, no endothermic peak was observed in the curve of SPI-Cur-PE nanocomplexes, it indicated that Cur was encapsulated in an amorphous state, which was consistent with the FT-IR and XRD analysis results.

### 3.3 Stability results

#### 3.3.1 Thermal stability

**Fig. 3:**
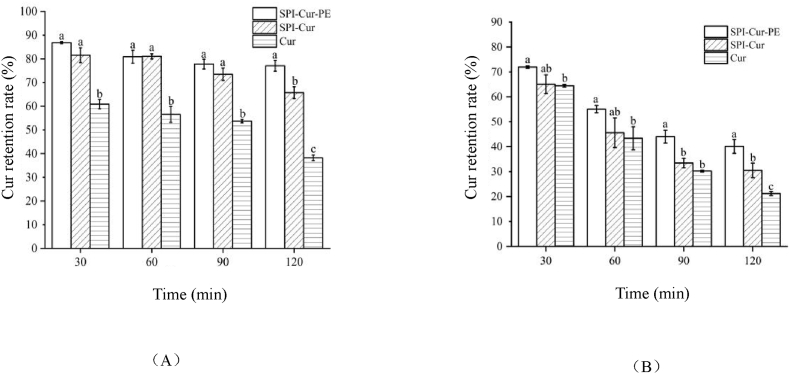
Stability of SPI-Cur-PE nanocomplexes.

The Cur, SPI-Cur, and SPI-Cur-PE were studied about thermal stability at 85 °C according to the DSC analysis, and the results were shown in Fig. 3 (A). As could be seen, the Cur retention rate in free Cur, SPI-Cur, and SPI-Cur-PE all decreased with prolonged heating time. Cur was unstable and degraded at 85 °C of heat treatment. The retention rate of Cur was 60.9% after 30 min of heat treatment. The retention rate dropped down to 38.2% at 120 min. Meanwhile, the stability of Cur in SPI-Cur and SPI-Cur-PE was significantly improved. The retention rate of Cur was 65.7% in SPI-Cur and 77.0% in SPI-Cur-PE at 120 min after thermal treatment, respectively. It demonstrated that thermal stability of Cur after SPI and PE encapsulating was enhanced, and similar result had been reported in the study of Zhan et al. (Zhan et al., 2020).

#### 3.3.2 Photostability

The Cur retention rates of free Cur, SPI-Cur and SPI-Cur-PE were determined at 25 °C with light intensity of 100 W/m^2^ to investigate the photostability of Cur combined with SPI and PE, and the results were shown in Fig. 3 (B). The figure shown the retention of Cur in SPI-Cur and SPI-Cur-PE decreased with the increase of illumination time. At light intensity of 100 W/m^2^, the free Cur was degraded rapidly, with Cur retention below 50% at 60 min and 21.2% at 120 min. While the photostability of SPI-Cur and SPI-Cur-PE increased significantly. The retention rate of Cur in SPI-Cur and SPI-Cur-PE was 30.5% and 40.1% after 120 min of illumination, respectively. It may be due to the causation that SPI and PE could alter the chemical stability of the reaction group in Cur by forming complex with Cur, thereby affecting the photostability (Xu et al., 2020).

### 3.4 In vitro release properties

**Fig. 4:**
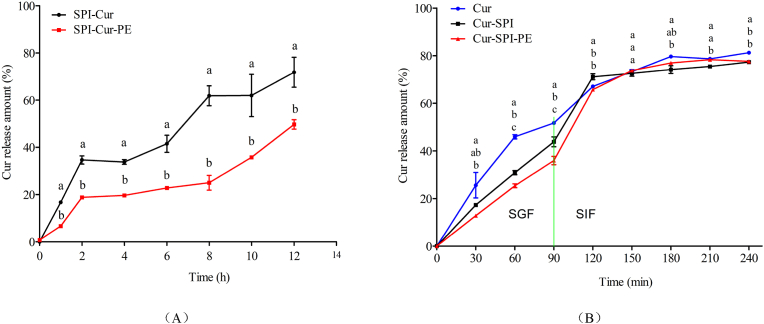
In vitro release and simulated gastrointestinal release.

The kinetics of Cur release in the SPI-Cur and SPI-Cur-PE nanocomplexes were investigated, and the results were shown in Fig. 4 (A). The release rate of Cur in the SPI-Cur nanocomplexes was 71.8% after 12 h, whereas the release rate of Cur in the SPI-Cur-PE nanocomplexes was 48.3%. Compared with SPI-Cur, the SPI-Cur-PE nanocomplexes formed by PE wrapping could slow down the release rate of Cur and achieved more obvious slow-release effect. The slowdown of the release rate may be because the “nuclear-shell” structure formed by SPI and PE, which could effectively limit the diffusion of encapsulated Cur molecules (Mirpoor et al., 2017).

### 3.5 Simulated gastrointestinal release

The simulated gastrointestinal release curves of free Cur, SPI-Cur and SPI-Cur-PE were shown in Fig. 4 (B). The Cur release rates of free Cur, SPI-Cur, and SPI-Cur-PE nanocomplexes were 51.7%, 43.8%, and 36.0%, respectively after 90 min of SGF digestion. In contrast, SPI-Cur released more slowly than free Cur in the SGF. It may because the SPI could protect lipid-soluble substances while reducing the release in the stomach (S. Wang et al., 2020). A significant increase release rate phenomenon could be observed in Fig. 4 (B) when the SPI-Cur and SPI-Cur-PE were transferred from the SGF to the SIF. The release rate of the SPI-Cur-PE increased from 36.0% to 65.7%, possibly due to the PE dissolution coated on the composite nanoparticle surface (Hu et al., 2015). The release rate of Cur in SPI-Cur-PE was consistently lower than that of the free Cur during the simulated gastrointestinal release. The release rates of Cur in free Cur, SPI-Cur, and SPI-Cur-PE were 81.3%, 77.4%, and 77.3%, respectively at 240 min. From the above results, it was clear that compared with free Cur, SPI-Cur and SPI-Cur-PE nanocomplexes was less dissolved and decomposed in the stomach, which could be more effectively released in the intestine.

### 3.6 The antioxidant activity in vitro

**Fig. 5:**
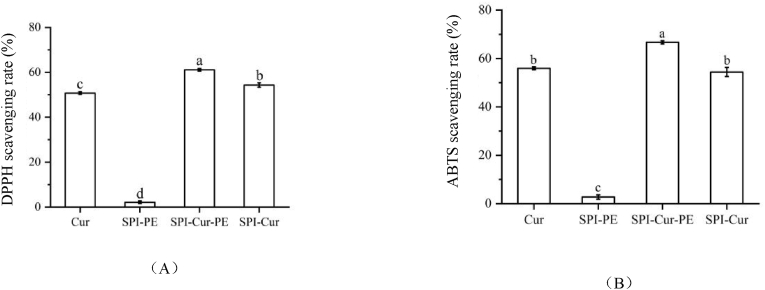
Antioxidant activity of nanocomplexes in vitro.

The in vitro antioxidant activity of free Cur, SPI-Cur and SPI-Cur-PE were evaluated by DPPH and ABTS radical scavenging tests, and the results were shown in Fig. 5. It could be seen that the antioxidant activity of the SPI-Cur-PE was significantly higher than that of the free Cur and SPI-Cur. On one hand, the reason may be that the water solubility and dispersion of the SPI-Cur-PE increased, thus improving the antioxidant activity. On the other hand, it could be seen that the SPI-PE also had a weak antioxidant activity, which may enhance the antioxidant capacity of the SPI-Cur-PE nanocomplexes. Huang et al. (X. Huang et al., 2016) and Wu et al. (Y. Wu and Wang, 2017) encapsulated Cur with core-shell protein-polysaccharide nanoparticles and self-assembled casein-dextran conjugate micelles, respectively, and similar results appeared in their researches.

## 4 Conclusion

The SPI-Cur-PE nanocomplexes prepared successfully by using SPI and PE in this study. The encapsulated of Cur with SPI and PE improved its photostability and thermal stability. SPI-Cur-PE shown lower releasing speed than SPI-Cur in vitro release test. In addition, the release rate of SPI-Cur-PE in SGF was lower than that of SPI-Cur. The SPI-Cur-PE represented significantly scavenging activity of ABTS and DPPH radicals than that of the SPI-Cur and free Cur. In general, the SPI-Cur-PE nanocomplexes had decent photostability, thermal stability, higher antioxidant activity and slower release in vitro performance than free Cur, which had development value and application prospects in the food field.

## CRediT authorship contribution statement

**Lijuan Fu:** Writing – original draft, Writing – review & editing, Formal analysis. **Suo Tan:** Conceptualization, Data curation, Methodology, Investigation, Software, Formal analysis. **Ruiru Si:** Validation. **Yueyue Qiang:** Validation. **Hang Wei:** Validation. **Biao Huang:** Validation. **Mengzhu Shi:** Validation. **Ling Fang:** Validation. **Jianwei Fu:** Supervision, Resources, Funding acquisition. **Shaoxiao Zeng:** Supervision, Resources.

## Declaration of competing interest

None.

## Data Availability

Data will be made available on request.
