# Supplementary material for: Characterization, stability and antioxidant activity of curcumin nanocomplexes with soy protein isolate and pectin
Source: Curr Res Food Sci. 2023 Jun 7;6:100530. doi: 10.1016/j.crfs.2023.100530 (PMC10290990; doi:10.1016/j.crfs.2023.100530)
Supplement: Multimedia component 1 [file mmc1.docx]

| （A） 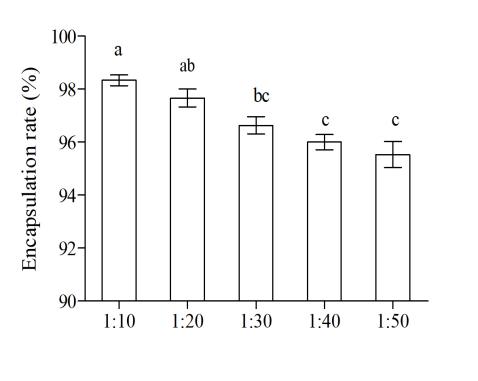 The volume ratio of Cur :SPI | （B） 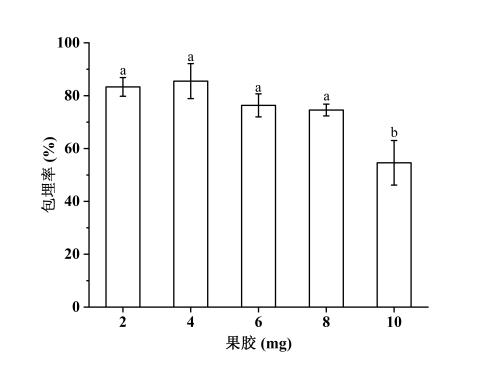 PE (mg)  Encapsulation rate (%) |
| --- | --- |
| （C） 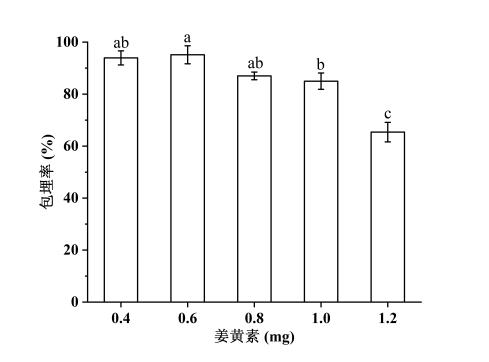 Cur (mg)  Encapsulation rate (%) | （D） 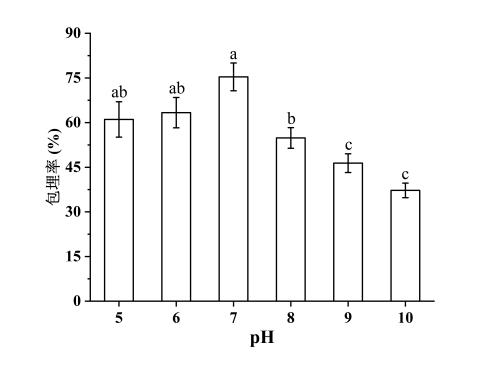 pH  Encapsulation rate (%) |
| **Supplement Fig. 1. Effect of volume ratio of Cur : SPI (A), amount of PE (B) & Cur (C) added and pH (D) on encapsulation rate**  Different letters (a-c) represent significant differences | |

| **Supplement Table 1. Orthogonal experimental design and results** | | | | |
| --- | --- | --- | --- | --- |
| Treatment group | PE added (mg) | Cur added (mg) | pH | Embedding rate (%) |
| 1 | 2.0 | 0.4 | 5.0 | 90.14 |
| 2 | 2.0 | 0.6 | 6.0 | 88.27 |
| 3 | 2.0 | 0.8 | 7.0 | 89.72 |
| 4 | 4.0 | 0.4 | 6.0 | 92.91 |
| 5 | 4.0 | 0.6 | 7.0 | 97.81 |
| 6 | 4.0 | 0.8 | 5.0 | 78.54 |
| 7 | 6.0 | 0.4 | 7.0 | 84.46 |
| 8 | 6.0 | 0.6 | 5.0 | 82.56 |
| 9 | 6.0 | 0.8 | 6.0 | 87.05 |
| K1 | 268.13% | 267.51% | 251.24% | / |
| K2 | 269.26 % | 268.64% | 268.23% | / |
| K3 | 254.07 % | 255.31% | 271.99% | / |
| k1 | 89.38% | 89.17% | 83.75% | / |
| k2 | 89.75% | 89.55% | 89.41% | / |
| k3 | 84.69% | 85.10% | 90.66% | / |
| R | 5.06% | 4.44% | 5.66% | / |
